# Let’s Make Life Easier for Health Workers, Not More Complicated

**DOI:** 10.9745/GHSP-D-22-00020

**Published:** 2022-02-28

**Authors:** Stephen Hodgins

**Affiliations:** a Editor-in-Chief, Global Health: Science and Practice Journal, and Associate Professor, School of Public Health, University of Alberta, Edmonton, Alberta, Canada.

See related article by Moukénet et al.

The article by Moukénet et al. in this issue of GHSP, draws attention to a practical problem faced by health workers seeking to deliver sick child care rationally at the primary health care level. In this article, the specific challenge was the provision of seasonal presumptive malaria treatment to young children in Chad. As is the case in most countries where *Plasmodium falciparum* malaria remains a problem of public health importance, children aged younger than 5 years (as well as pregnant women) are targeted for both preventive and curative malaria services. This prioritization is reflected in commodity procurement and distribution and in clinical protocols to be used by health workers.

But what looks rational for a central-level program planner or manager can look decidedly less so for a health worker charged with providing care to a sick child presenting at their health post. Whether the child in front of them is aged 4 years and 11 months old or 5 years and 1 month, that child faces similar risks from the illness and derives similar benefits from the treatment the health worker could provide. Thankfully, in many instances, health workers do not arbitrarily refuse treatment to children falling a little beyond what’s specified in their treatment protocols.

For central-level planners and program managers (and those providing them technical assistance), keeping things simple makes their lives easier. But overly simplistic program design can make life more complicated for health workers and those they are trying to serve. The article by Moukénet et al.^1^ is a helpful reminder of the importance of checking to see what’s actually happening on the ground and, where necessary, making adjustments to our programs to better respond to that reality.
